# Intraoperative damage to surgical gloves during various operations on the musculoskeletal system: a multicenter study

**DOI:** 10.1007/s00402-020-03594-1

**Published:** 2020-08-29

**Authors:** Andreas Enz, Tanja Kostuj, Philipp Warnke, Katrin Osmanski-Zenk, Wolfram Mittelmeier, Annett Klinder

**Affiliations:** 1 Orthopädische Klinik und Poliklinik, Universitätsmedizin Rostock, Rostock, Germany; 2 Orthopädisch-Traumatologisches Zentrum, St. Marien-Hospital Hamm, Hamm, Germany; 3 Institut für Medizinische Mikrobiologie, Virologie und Hygiene, Universitätsmedizin Rostock, Rostock, Germany

**Keywords:** EN 455-1, Damage, Orthopedic surgery, Surgical side infection, Glove, Lesions

## Abstract

**Introduction:**

Various orthopedic surgical procedures cause mechanical stress for gloves. In some cases, sharp-edged objects impact on the glove surfaces. The systematic description of lesions is still missing.

**Methods:**

2289 gloves from 409 surgeries [primary hip and knee arthroplasties (PA), revisions arthroplasties (RA) and arthroscopic shoulder, hip and knee surgery (AY)] from 3 clinics were examined for lesions using water tightening test according to the European norm EN 455-1.

**Results:**

Arthroscopies showed the lowest rate of operations with damaged gloves (6.9%). Depending on clinic, 32.7% and 59.2% of PA surgeries generated damaged gloves, while in RA, these numbers rose to 76.0% and 72.8%, respectively. In PA and RA, the most affected finger was the index finger, whereas in arthroscopies, more damage occurred on the middle finger and the thumb. The size of the lesions was rather small with the vast majority being 1 mm or 2 mm in size.

**Conclusion:**

All investigated interventions led to glove lesions. With increasing mechanical stress, the number of glove defects increased. EN 455 does not account for the intraoperative tear risk. Stricter requirements for gloves should be introduced. Glove change intervals should be defined and implemented, and new materials should be developed.

## Introduction

Surgical gloves with their thin layer of latex are the only mechanical barrier between the patient and the operating team and are intended, apart from various (constructional-) technical requirements in an operating theatre, to protect both sides against infection [1, 2]. The sterile latex surgical glove is used in a variety of disciplines, for example in gastroscopy, cardiac catheter interventions or in invasive and minimally invasive orthopedic and traumatological procedures [3, 4]. According to the European Committee for Standardization (CEN) standard EN 455, the gloves produced are randomly tested for waterproofness (EN 455-1) by means of a water tightening test (1L water for 2–3 min) and tear resistance (EN 455-2) with a maximum weight of 9 N as standard. The defined acceptable quality limit (AQL) for surgical gloves was 1.5, which means that for 50 tested latex gloves, 2 gloves may be damaged without the production batch having to be discarded [5]. In the draft standard (preEN 455-1:2019), the AQL has been adjusted to 0.65 [6] and was adopted in the new version EN 455-1:2020 [7]. Nevertheless, the currently valid standard is significantly less stringent as compared to the testing of other latex products, such as for example condoms (AQL 0.25) [8]. Mechanical loads such as shear force, repetitive movements and sharp-edged surfaces are not taken into consideration when testing surgical gloves [5]. No specifications regarding minimum thickness, tensile strength and elongation at break are given. The aim of this study was to investigate whether operations with different mechanical stress have an influence on the frequency and type of perforation. A total of 2289 gloves were tested and evaluated by means of a water tightening test (EN 455-1) after primary and revision endoprosthetics on knee and hip, as well as after arthroscopic surgery.

## Method

### Clinics and operations

At the Clinic for Orthopedics and Trauma Surgery, Catholic Hospital Bochum, University Hospital of the Ruhr-University Bochum and the Clinic for Orthopedics and Trauma Surgery Clinic Lippe in Lemgo (BuL) surgical gloves from primary hip and knee endoprostheses as well as hip and knee revision arthroplasties were examined and compared to the data of the Orthopedic Clinic and Policlinic of the University Medical Center Rostock (UMR) which were collected from primary hip and knee arthroplasties, hip and knee revision arthroplasties. Additionally, gloves retrieved from arthroscopies (hip, knee and shoulders), were collected at UMR.

### Gloves

From July 2016 until October 2019, 827 surgical gloves were collected at the Clinic for Orthopedics and Trauma Surgery, Catholic Hospital Bochum, University Hospital of the Ruhr-University Bochum and the Clinic for Orthopedics and Trauma Surgery Clinic Lippe in Lemgo (BuL) and subsequently compared to an existing database from the Orthopedic Clinic and Policlinic of the Rostock University Medical Center about glove damage whose data were partly reported previously [9, 10]. In total, 2289 surgical gloves from 409 surgeries, including 153 primary endoprostheses implantations, 155 revision arthroplasties and 101 arthroscopic interventions, were examined. The number of gloves used, number of gloves per operation, type of operation (knee, hip or shoulder), duration of the operation, type of surgeon (according to Endocert certification) and the use of bone cement or the removal of bone cement were documented. All operations were performed with two pairs of gloves in the so-called double-gloving procedure. Sterile, powder-free latex gloves for single use (ProtexisTM, Cardinal Health, Dublin, Ohio, USA (AQL 0.65); Vasco^®^ OP eco, B.Braun, Melsungen, Germany (AQL 0.65); Neolon^®^ 2G surgical gloves, Medline Industries, Inc, Northfield, USA (AQL 0.65); Biogel Eclipse, Mölnlycke Health Care, Gothenburg, Sweden (AQL 0.65) and Sempermed supreme, Sempermed/Semperit, Vienna, Austria (AQL 0.65) were investigated.

### Method of investigation

Gloves from endoprosthetic interventions were changed intraoperatively as a routine procedure before implantation of the endoprosthesis parts, contact with cement, in the case of obvious damage and after 2 h (for more details, see Text box A). The gloves of the leading surgeon were collected and packed for each individual operation and the data relevant for evaluation were documented. For operations without change of gloves (arthroscopies), the gloves were collected after finishing the operation. The examination was carried out in the UMR laboratory. Gloves from BuL were packed separately for each operation, free of pressure points and sent to Rostock for evaluation. As control, the influence of glove undressing was tested on 50 gloves without surgery. 50 gloves without surgery were tested after postage (3 days duration). The evaluation was performed according to EN 455—Medical gloves for single use part 1, method for testing for freedom from holes with water tightening test [5, 10]. The localization of the damage was determined, size and dimension were measured with a plastic goniometer (Kirchner & Wilhelm GmbH & Co. KG, Asperg, Germany) and the lesion configuration was recorded with microscopes (Laser Scanning Microscope VK-S1100 and Digital Microscope VHX-6000, Keyence, Germany).

**Text box A:** Intraoperative glove change algorithm

Operation	Glove change		
Primary arthroplasty	implantation cup	Implantation stem/components	Obvious damage, cement contact, after 2 h surgery
Revision arthroplasty	After mechanical stress	Implantation of the definitive implants	Obvious damage, cement contact, after 2 h surgery
Arthroscopy	Duration over 1 h		Obvious damages

### Statistics

The collected data were analyzed using SPSS Statistics Package Version 22 (IBM Corp., New York, USA). Descriptive statistics were calculated for continuous and categorical variables. Continuous variables are displayed as mean values and standard deviations (SD) as well as median and range as most of the data were not normally distributed. Categorical factors are shown as frequency (*n*) with percentages in brackets. Testing for differences of categorical factors between different types of operations was done by the exact Fisher test (two categories) or by Pearson’s chi-square test (more than two categories). Testing for differences in continuous variables between different types of operations was performed using Kruskal–Wallis test. The significance level was set at *p* < 0.05.

### Ethics vote and data privacy

Ethics approval for the study was granted by the local ethics committee of the Rostock University Medical Center (registration number: A2016-0112) and data protection requirements were observed.

## Results

### General patient data and surgical method

The demographic data of the 409 participating patients are listed in Table 1. A total of 2289 surgical gloves were collected, 540 gloves from 104 primary arthroplasty operations, 669 gloves from 100 revision arthroplasty operations and 251 gloves from 101 arthroscopy interventions. A total of 827 gloves from the clinics in Bochum and Lemgo were analyzed, 346 gloves were used in 49 primary endoprostheses and 481 in 55 revision operations, of which 226 were inner gloves and 601 were outer gloves.

**Table 1:** Descriptive analysis of patient demographic data

	Patients with primary arthroplasty		Patients with revision arthroplasty		Patients with arthroscopy
	UMR	BuL	UMR	BuL	UMR
Patient data					
Number of patients recruited (*n*)	104	49	100	55	101
Male [*n*, (%)]	49 (47.1)	16 (32.7)	49 (49.0)	24 (43.6)	47 (46.5)
Female [*n*, (%)]	55 (52.9)	33 (67.3)	51 (51.0)	31 (56.4)	54 (53.5)
Age in years [M ± SD; MD (range)]	68.1 ± 11.4; 72 (20–84)	74.5 ± 11.9; 73 (34–98)	68.9 ± 10.4; 71.5 (22–84)	76.7 ± 10.8; 79 (47–93)	47.6 ± 16.3; 51 (12–82)
Body mass index [M ± SD; MD (range)	29.9 ± 5.5; 29.3 (19.2–49.3)	28.4 ± 5.0; 27.9 (15.8–39.2)	29.2 ± 5.4; 29.0 (17.6–44.4)	28.94 ± 5.8; 28.1 (20.8–50.7)	27.9 ± 4.9; 27.1 (16.8–42.8)

### Comparison of intraoperative lesions

Table 2 shows the comparison of the three types of surgery, PA, RA and AY, as well as the damage on the other centers (cumulative). While gloves were damaged the least (6.9%) in AY, the number of operations with glove damage increased to 32.7% in PA-UMR and 59.2% in PA-BuL. In RA-BuL, 72.7% and RA-UMR 76.0% of the surgeries were found to have damaged gloves. While the overall percentage of damaged gloves in PA and RA ranged from 10.9 to 25.0% in both centers, a detailed analysis of the gloves collected in the BuL clinics showed that inner gloves were with 6.2% less damaged than outer gloves (19.0% damaged/total number of gloves).

**Table 2:** Statistical analysis of surgical data with regard to the occurrence of glove damage of the entire surgery

	Primary arthroplasty		Revision arthroplasty		Arthroscopy	*p *value^*^
	UMR	BuL	UMR	BuL	UMR	
OP-specific data						
Total number of gloves used (*n*)	542	346	669	481	251	2.289 overall
Number of surgical gloves						
Undamaged [*n*, (%)]	483 (89.1)	300 (86.7)	502 (75.0)	399 (83.0)	244 (97.2)	< 0.0001^+^
Damaged [*n*, (%)]	59 (10.9)^a, b, c^	46 (13.3)^a, b, c^	167 (25.0)^b^	82 (17.0)^a, b^	7 (2.8)^c^	
Number of operations						
Without damaged gloves [*n*, (%)]	70 (67.3)	20 (40.8)	23 (23.0)	15 (27.3)	94 (93.1)	< 0.0001^+^
With damaged gloves [*n*, (%)]	34 (32.7)^a^	29 (59.2)^b^	77 (77.0)^b^	40 (72.7)^b^	7 (6.9)^c^	
Average number of damaged gloves per surgery [M ± SD; MD (range)]	5.2 ± 2.1; 4 (2–12)^a^	7.1 ± 2.4; 6 (4–16)^b^	6.7 ± 3.0; 6 (2–14)^b^	8.7 ± 4.5; 8 (4–28)^b^	2.5 ± 0.9; 2 (2–4)^c^	< 0.0001^^^
Average number of damaged gloves per surgery [M ± SD; MD (range)]	06 ± 0.9; 0 (0–3)^a^	0.9 ± 1.0; 1 (0–3)^a, b^	1.7 ± 1.6; 1 (0–8)^b^	1.5 ± 1.4; 1 (0–7)^b^	0.1 ± 0.3; 0 (0–2)^c^	
						< 0.0001^^^
Operated joint						
Shoulder [*n*, (%)]	–		–		20 (19.8)	
Hip [*n*, (%)]	77 (74.1)^a^	31 (63.3)^a,b^	74 (74.0)^a^	27 (49.1)^b^	6 (5.9)^c^	
Knee [*n*, (%)]	27 (25.9)^a^	17 (34.7)^a,b^	26 (26.0)^a^	28 (50,9)^b^	75 (74.3)^c^	< 0.0001^+^
Ankle joint [*n*, (%)]		1 (2.0)				
Surgery by						
Main operator [*n*, (%)]	72 (6.2)	45 (91.8)	94 (94.0)	54 (98.2)	84 (86.1)	< 0.0001^+^
Surgeon in training [*n*, (%)]	32 (30.8)^a^	4 (8.2)^b^	6 (6.0)^b^	1 (1.8)^b^	17 (13.9)^a,b^	
Use of bone cement						
Cemented [*n*, (%)]	73 (70.2)	21 (42.9)	52 (52.0)	23 (41.8)	–	0.0008^+^
Cementless [*n*, (%)]	31 (29.8)^a^	28 (57.1)^b^	48 (48.0)^b^	32 (58.2)^b^	–	
Removal of bone cement						
Yes [*n*, (%)]	–	–	49 (49.0)	24 (43.6)	–	0.6143̊
No [*n*, (%)]	–	–	51 (51.0)	31 (56.4)	–	
Duration of surgery in min [M ± SD; MD (range)]	79.3 ± 23.3; 76 (28–140)^a^	91.6 ± 25.5; 88 (52–195)^a, b^	116.8 ± 48.4; 112.5 (30–310)^b^	129.7 ± 62.5; 118 (23–323)^b^	40.7 ± 20.5; 37 (15–112)^c^	< 0.0001^^^

^^^Kruskal–Wallis test

^+^Chi-square test

^°^Fisher’s exact test for 2 groups

*Total comparison of all 5 groups using Kruskal–Wallis test, Chi-square test or Fisher’s exact test

^a,b,c^Groups with different small letters show significant differences (*p* < 0.05), groups with the same small letters do not show significant differences

### Gloves and operating time

The highest number of gloves per surgery was spent in RA, the intervention with the longest duration of surgery (Table 2). The correlation between number of damaged gloves and duration of surgery was significant in RA (BuL *r* = 0.363, *p* = 0.007; UMR *r* = 0.208, *p* = 0.037), but not in primary endoprosthetics (BuL *r* = 0.087 *p* = 0.553, UMR r = 0.093 *p* = 0.346). The use of bone cement for fixation of the implant in PA and RA had no influence on the number of damaged gloves in the center of Rostock. In contrast, cement removal in BuL showed a significant influence on the lesion rate of the gloves (*p* = 0.026) in RA. Overall, there was a correlation in the damage rate between operations with and without cement removal (*p* = 0.054), but not between uncemented and cemented implants (*p* = 0.488).

### Location and size of the glove lesions

**Fig. 1:**
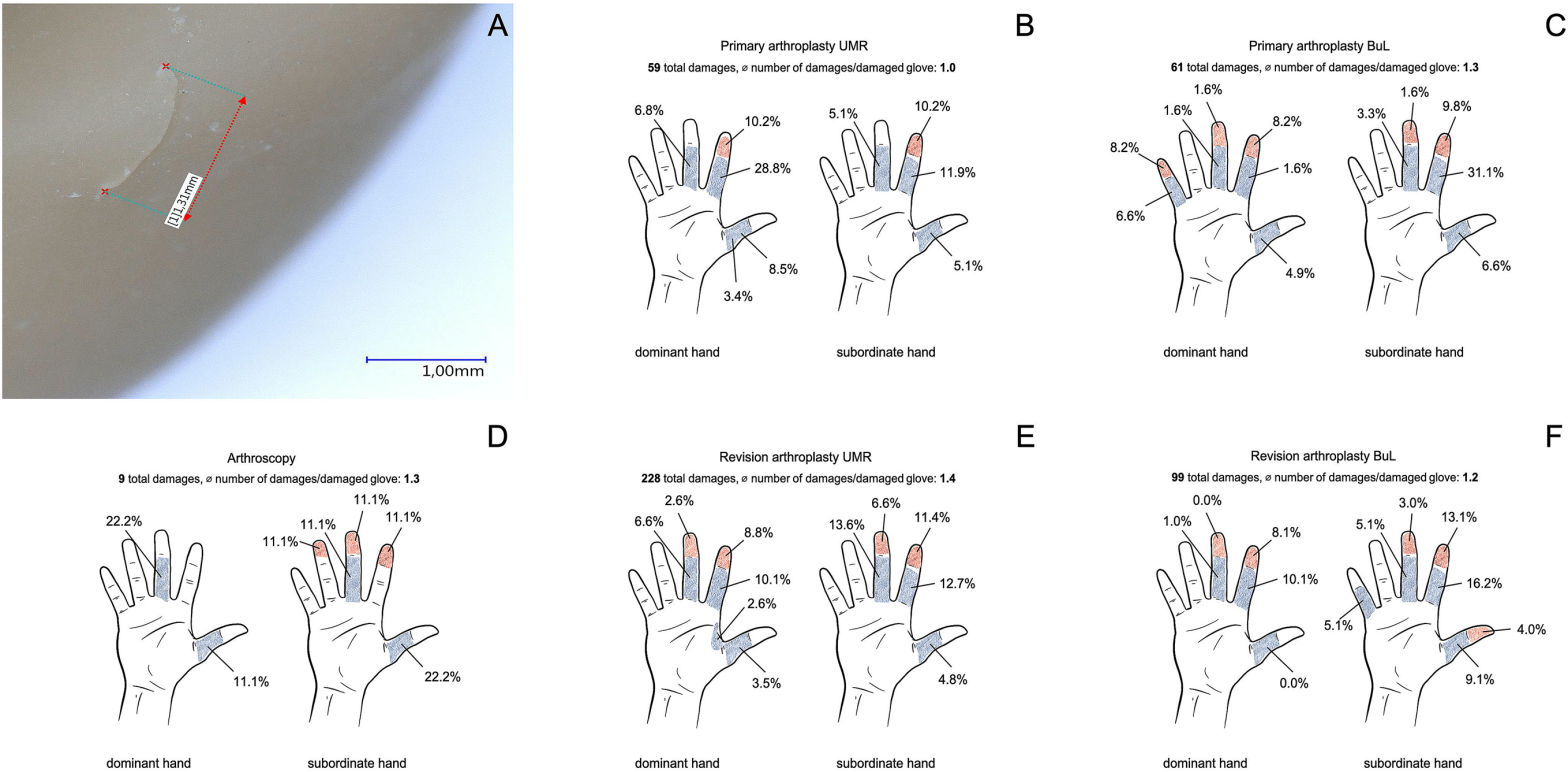
**a** typical lesion pattern of a glove damage (100 × magnification), **b**–**f** distributions of the damages with frequencies in % and number of damages per damaged glove according to the examined operation and clinic

Significant differences in the position of glove damage were found for the different types of surgery. The lesion rate for PA on the index finger and index fingertip was 61.1% (UMR) and 50.7% (BuL), for RA 43.0% (UMR) and 47.5% (BuL). In AY, thumbs were affected in 33.3% and middle fingers in 55.0% of cases. In gloves used at the UMR, the damage in PA was in the area of the dominant hand (61.0%), in RA rather in the area of the subordinate hand (59.6%). Gloves from operations at BuL displayed glove damage more frequently in the area of the subordinate hand in PA and RA (Fig. 1b, c). For a detailed analysis, see Table 3 and Fig. 1 (b–f). In AY, 66.7% of the damage was in the area of the subordinate hand, distributed over the thumb (22.2%), index fingertip (11.1%), middle finger and middle fingertip (22.2%) and tip of the ring finger (11.1%). The dominant hand showed only a third of the damage, located almost exclusively in the thumb and middle finger area (11.1% and 22.2%, respectively) (Fig. 1d). The dimensions of glove damage ranged from ≤ 1 mm to more than 5 mm of size. The size of the lesions in the gloves varied significantly between PA, RA and AY as well as among the centers (Table 3).

**Table 3:** Percentage of occurrence of certain (A) localizations and (B) sizes of damage in relation to the total number of damages

	Primary arthroplasty				Revision arthroplasty				Arthroscopy	
	UMR		BuL		UMR		BuL		UMR	
Total number of damages Ø damages/damaged glove	59 1.0		61^a^ 1.3		228^a^ 1.4		99^a^ 1.2		9^a^ 1.3	
	DH	SH	DH	SH	DH	SH	DH	SH	DH	SH
(A) Localizations										
Thumbs	8.5	5.1	4.9	6.6	3.5	4.8	0.0	9.1	11.1	22.2
Thumb tip	0.0	0.0	0.0	0.0	0.9	1.8	1.0	4.0	0.0	0.0
Thumb/Rasp marks	3.4	0.0	0.0	0.0	0.0	0.0	0.0	0.0	0.0	0.0
Index finger	28.8	11.9	1.6	31.1	10.1	12.7	10.1	16.2	0.0	0.0
Index fingertip	10.2	10.2	8.2	9.8	8.8	11.4	8.1	13.1	0.0	11.1
Middle finger	6.8	5.1	1.6	1.6	6.6	13.6	1.0	5.1	22.2	11.1
Middle fingertip	0.0	0.0	1.6	3.3	2.6	6.6	0.0	3.0	0.0	11.1
Ring finger	0.0	0.0	0.0	1.6	0.0	1.3	1.0	2.0	0.0	0.0
Ring fingertip	0.0	0.0	0.0	0.0	0.0	0.0	0.0	0.0	0.0	11.1
Little finger	0.0	0.0	6.6	0.0	0.0	0.0	2.0	5.1	00	0.0
Little fingertip	0.0	0.0	8.2	0.0	0.0	0.4	0.0	1.0	0.0	0.0
Palm	1.7	3.4	6.6	1.6	3.1	2.6	3.0	3.0	0.0	0.0
Dors FA/back of the hand	0.0	0.0	0.0	1.6	0.4	2.2	5.1	1.0	0.0	0.0
Palm FA	0.0	0.0	1.6	0.0	1.8	0.4	1.0	3.0	0.0	0.0
Between thumb and index finger	0.0	1.7	0.0	0.0	2.6	1.8	0.0	1.0	0.0	0.0
Index finger/middle finger	0.0	1.7	1.6	0.0	0.0	0.0	1.0	0.0	0.0	0.0
Between index finger and middle finger	1.7	0.0	0.0	0.0	0.0	0.0	0.0	0.0	0.0	0.0
Percent per DH/SH	61.0	39.0	42.6	57.4	40.4	59.6	33.3	66.7	33.3	66.7

Total number of damages mm										
(B) Sizes of damage (mm)										
1	5.1	6.8	23.0	37.7	8.8	23.7	11.1	39.4	11.1	44.4
2	22.0	13.6	8.2	11.5	12.7	18.0	12.1	14.1	11.1	11.1
3	15.3	10.2	3.3	0.0	5.3	6.6	2.0	8.1	11.1	0.0
4	13.6	5.1	0.0	3.3	3.5	4.4	4.0	1.0	0.0	11.1
5	1.7	3.4	3.3	3.3	1.8	3.5	2.0	2.0	0.0	0.0
6	1.7	0.0	0.0	1.6	3.9	1.8	0.0	0.0	0.0	0.0
7	0.0	0.0	1.6	0.0	0.4	0.4	0.0	0.0	0.0	0.0
8	0.0	0.0	1.6	0.0	0.4	0.4	0.0	0.0	0.0	0.0
9	0.0	0.0	0.0	0.0	0.9	0.0	0.0	0.0	0.0	0.0
10	0.0	0.0	0.0	0.0	1.3	0.4	0.0	1.0	0.0	0.0
> 10	1.7	0.0	1.6	0.0	0.9	0.4	2.0	1.0	0.0	0.0

Data in %

*DH* dominant hand, *SH* subordinate hand

^a^The total number of damages may differ from the total number of damaged gloves because in some cases several damages per glove were found.

## Discussion

In the present study, it was shown that even arthroscopic procedures with little mechanical stress can cause severe damage to the gloves. With increasing mechanical stress, gloves are subject to a higher risk of lesions. It is, therefore, recommended to use the so-called double gloving (DG), i.e. to wear a second pair of gloves on top of the first pair [2]. The literature indicates that the practice of the DG is not practiced uniformly [11], lack of sensitivity in the finger is considered one of the main reasons among surgeons when dispensing with a second pair of gloves (single gloving) [12]. In endoprosthetics, the use of two pairs of gloves is an established standard [13], which is confirmed as necessary by the data of this study and by others [9, 14, 15].

### Mechanical stress and operating time

With increasing mechanical stress, the rate of damage to the material increases, resulting in lesions in the gloves. Harnoss et al. showed in septic laparotomies with low mechanical stress that damage to the glove remains mostly unnoticed and in almost 5% of cases bacteria can pass through these lesions [16]. It became apparent, that after perforation, bacterial strains were found in the wound infection which were identical to those found on the hand of the performing surgeon [17]. Glove lesions can pose a risk of surgical wound infections. The data of our study prove that, in addition to the mechanical stress, the duration of the operation also has an influence on the number of glove lesions. The number of glove damage increases with the duration of the operation [14], which may result in increased translocation of bacteria and thus could be an explanation for the higher infection rates in RA [18, 19]. Using gloves with indicator system, perforations could be noticed faster and a change of gloves could take place [20]. Definite algorithms for changing gloves even without obvious damage should be established and integrated into the surgical hygiene regulations to reduce infections by microperforation [15, 16]. The amount of gloves used in RA interventions was significantly higher than in PA and AY, due to the glove change algorithm of the participating clinics (see Text box A). The algorithm takes into account the increased risk of glove damage during mechanically demanding procedures with longer operating times, which is confirmed by the higher number of gloves worn. The literature confirms increased perforation rates in gloves of RA [21].

The use of PMMA bone cement is considered to be a factor for increasing damage rates. Statistically, there was no significant difference in the number of damaged gloves for uncemented and cemented implants, but there was a higher risk of damage when cement was removed during RA.

### Location of lesions

The index finger of the surgeon’s dominant and subordinate hand proved to be particularly prone to lesion, especially in RA [9, 10, 22]. This is in line with the findings of other research groups [21, 23]. The shift of lesions from the dominant hand to the subordinate hand in RA, which is present in UMR, can be explained by an increased tactile behavior of the subordinate hand. Different surgical techniques and patient positioning can play a role in this. A comparison of the three endoprosthetic sites showed that there were significant differences in the positions and sizes of the lesions, as well as differences in dominant and subordinate hand. While the number of surgeries in which damage occurred was almost the same for RA in the centers, there was a significant difference in PA-UMR and PA-BuL. With equal qualification and experience of the surgeons at the centers, the variations might be explained by different glove manufacturers, different implant designs (screw cups) and surfaces, instruments (use of slide hammer), different surgical accesses and different patient positioning. Especially, the differences in the lesion frequency in gloves of the subordinate hand from operations at BuL can be explained by local factors. For example, one of the surgeons in BuL claims to use both hands equally, and after a fracture during childhood, a shortening of a finger occurred with the glove not fitting properly in that area. The influence of the design of the implantation instruments on the perforation rate of surgical gloves has not yet been investigated. Only a few studies are available on sutures [24].

In contrast, fewer lesions were found during arthroscopic procedures, which can be explained by the missing of lesions-risk such as open-rotating instruments (reamers, drills) [25]. In this study, the middle finger and thumb area were significantly more often affected. In the literature, the damage in arthroscopy is mainly attributed to the index finger [24, 26]. The discrepancy in results may be due to different knotting techniques used by surgeons and suture materials from different manufacturers. In an in vitro experiment with various arthroscopic suture materials, lesions in the index finger area occurred more often [24]. However, the experimental set-up shown by Martinez et al. is missing resilient elements corresponding to the soft tissue structures of the patient. Dynamic saw-like movements of the suture material additionally damage the glove and, depending on the knotting technique, explains accumulated lesions to other parts of the glove.

### Glove leakage detection methods and the EN 455 standard

To date, the water tightening test is the only required test to detect production associated leaks in gloves [6]. It is uncertain to what extent this test is suitable as the main detection method of glove injuries from the user’s point of view. In the literature, it has been shown that this test is inaccurate, microperforations are insufficiently detected [24, 27]. Test methods using electrical conductivity (ECT) show a significantly better resolution, especially for micro-lesions [27]. Further test protocols should be implemented in the test procedures for tightness, as already established for other latex products [8], despite significantly higher cost pressure for surgical gloves. It is questionable whether sterile latex gloves produced under this standard are sufficiently safe for surgical use.

### Limitations of the study

By lab-analyses, it was not possible to distinguish for each individual lesion whether it was caused initially by production process or during the operating procedure. No separate investigation was carried out for different producers and a distinction was made between latex and non-latex. Studies on translocation of bacteria through glove lesions were not performed. The influence on the postoperative wound infection rate as well as potential Infections of the staff were not examined.

## Conclusions

Mechanical stress led to lesions on sterile surgical gloves. With increasing stress, the number of lesions increased. Depending on the performed operating procedure, differences in the location of the lesions on the gloves were observed. Standards for the intraoperative change of gloves should be established according to the type of intervention and should be documented in hygiene regulations. The CEN specifications do not yet cover the high mechanical intraoperative stress on gloves. Mechanical testing and special procedures for testing for micro-lesions should be introduced in addition to new glove materials and design.
